# Occupational exposure to metal welding and cataract: A systematic review and meta‐analysis

**DOI:** 10.1111/aos.70066

**Published:** 2026-01-27

**Authors:** Jakob Bjerager, Esben Meulengracht Flachs, Martin Nissen Hermann, Jens Peter Ellekilde Bonde, Ingrid Sivesind Mehlum, Stinna Skaaby

**Affiliations:** ^1^ Department of Ophthalmology Rigshospitalet Glostrup Denmark; ^2^ Department of Occupational and Environmental Medicine, Bispebjerg Frederiksberg Hospital Copenhagen Denmark; ^3^ Faculty of Medical and Health Sciences University of Copenhagen Copenhagen Denmark; ^4^ National Institute of Occupational Health (STAMI) Oslo Norway; ^5^ Institute of Health and Society University of Oslo Oslo Norway

**Keywords:** cataract, occupational exposure, prophylaxis, ultraviolet radiation, welding

## Abstract

**Background:**

Metal welding generates ultraviolet radiation (UVR) of cataractogenic wavelengths, and UVR emitted during welding has been associated with cataract formation, but results from prior studies are conflicting.

**Methods:**

We conducted a systematic review and meta‐analysis of epidemiological studies addressing associations between occupational metal welding and cataract. The literature search was conducted on November 15, 2023, across seven literature databases. Studies comparing age‐adjusted occurrence of cataract among welders and control groups were included. Two reviewers extracted data, which were combined using random‐effects meta‐analyses.

**Results:**

We identified nine studies with a total of 5165 welders and 513 026 controls. There was considerable heterogeneity among studies (*I* ^2^ statistics: 39), and possible publication bias in favour of higher risk estimates. Stratified meta‐analyses revealed a summary OR of 1.22 (*n* = 3, 95% CI 0.79–1.90; *p* = 0.374) for cataract among welders in high‐income countries, whereas the OR was 2.95 (*n* = 9, 95% CI 1.68–5.19; *p* = 0.00017) in lower‐middle‐income countries.

**Conclusion:**

An increased risk of cataract among welders was found in studies from lower‐middle‐income countries, but not in studies from high‐income countries. The disparity could reflect differences in occupational safety adherence and study methodology and may also involve effect modification by cumulative solar UVR. Although a causal link between UVR from metal welding and cataract is biologically plausible, epidemiological evidence is still limited. More studies are needed to quantify the exact risk of cataract among welders in various populations.

## 1 INTRODUCTION

Welding of metal is a common industrial process in various sectors and is essential for construction, manufacturing, and maintenance activities. Worldwide, more than 10 million people are believed to be employed as welders, with an estimated additional 110 million workers involved in welding‐related activities (Guha et al., 2017). Welding entails exposure to numerous hazardous agents, including intense light, ultraviolet radiation (UVR), infrared radiation, and metal fumes. Prolonged and repeated exposure to these occupational hazards has been associated with various adverse health effects, including cataract formation (Tenkate, 1999).

Cataract, characterized by a clouding of the eye's lens, is a leading cause of severe vision impairment and blindness (WHO, 2014). More than 100 million individuals worldwide have an urgent and unmet need of cataract surgery, and the disease burden is expected to rise significantly, primarily due to ageing populations (Pesudovs et al., 2024). Although phacoemulsification cataract surgery is widely available in western countries and generally considered safe and efficacious, rates of pseudophakic retinal detachment are increasing (Nielsen et al., 2020), and the risk is higher in younger patients (Bjerrum et al., 2013). Welders may be subject to such risk due to cataract formation at an earlier age. Moreover, cataract surgery in developing countries is scarce; hence individuals affected by cataract at an early age, possibly including welders, may have to live with severe vision impairment or blindness for years (Khanna et al., 2011).

The exposure to UVR in welders has been thoroughly described in the literature and previously summarized (International Agency for Research on Cancer, 2018). UVR exposure is typically quantified through measures of irradiance (power per unit area, W/m^2^) or radiant exposure (J/m^2^), indicating the energy received per unit area over a specific time period (International Agency for Research on Cancer, 2018). In terms of which ocular structures are exposed, UVR wavelength is also of importance, as nearly all UVC radiation (100–280 nanometre) is absorbed in the cornea, while most of the remaining UVR is absorbed in the lens (Söderberg et al., 2016). Due to the highly variable emission of radiation during welding procedures, obtaining precise radiometric and spectroradiometric data is challenging (Tenkate, 2008). Exposure is dependent on the welding method, the material, and the intermittence (duration of welding) (Tenkate, 2008), along with the usage of protective equipment. The occupational exposure limit of 3 mJ/cm^2^, proposed by The International Commission on Non‐Ionizing Radiation Protection (ICNIRP), may be exceeded within seconds to minutes of welding (International Commission on Non‐Ionizing Radiation Protection, 2004). In addition to ultraviolet radiation, high‐heat welding such as gas metal and tungsten arc welding also generates nitrogen oxides including nitrogen dioxide (NO_2_), which is a potent oxidant, and chronic exposure has been implicated in cataract formation in population studies of ambient air pollution (Gayraud et al., 2025). Furthermore, several studies have found smoking to be associated with a higher prevalence of cataracts, and a higher incidence of cataract surgery, indicating a dose–response relationship (Nordström et al., 2025). A similar mechanistic link could be hypothesised for welding, which, like smoking, emits reactive oxygen species, free radicals, and redox‐active heavy metals.

While the association between welding and cataract formation has been widely studied, the existing evidence remains heterogeneous and inconclusive (West, 2007), underscoring the need for a comprehensive synthesis of available evidence. Understanding the possible association between welding and cataract formation is important for several reasons. Internationally, cataract is often not acknowledged as an occupational disease. In Denmark, cataract developed after occupational exposure to radiation is listed in the Occupational Disease Directory (The Danish Labour Market Insurance, n.d.) but no suspected cases have been reported to the Labour Market Insurance in recent years (Work Environment in Denmark, n.d.). Although occupational safety and health regulations are in place in most developed countries, less elaborate regulations or enforcement thereof might be found in some developing countries. In any case, robust scientific evidence may help to maintain or increase awareness of preventive measures in the workplace.

In light of these considerations, this systematic review and meta‐analysis aims to comprehensively evaluate and synthesize the existing literature on the association between metal welding and cataract formation.

## 2 MATERIALS AND METHODS

### 2.1 Literature search

The literature search for eligible studies was performed on 15 November 2023 and included four electronic bibliographic databases: Ovid MEDLINE, EMBASE, Cochrane Library and Web of Science, two grey literature databases (BIOSIS preview and Grey Literature) and reference lists of included studies. In addition, a web search engine (Google Scholar) was informally searched with broad search terms, such as “cataract and welding”. The literature search was originally performed to identify all possible occupational exposures associated with cataract (PROSPERO protocol 481 028). An information specialist aided in the search, and the full search string is available in Table A1.

### 2.2 Study selection

We included randomized controlled trials, cohort studies, case–control studies, and cross‐sectional studies that provided a relative or absolute estimate of the age‐adjusted association between welding and cataract. We excluded studies that did not control for age at least at the sampling level. No time restriction was placed. We only considered publications written in English or Scandinavian languages. Studies with abstracts only and publications with no original data were excluded.

### 2.3 Exposure and confounding variables

In the current study, we included all studies reporting occupational exposure to welding with either objective or subjective measures, such as self‐reports, job titles, expert ratings, or measurements. All types of welding were included. Studies lacking information on age or without age matching or other age control measures were excluded.

### 2.4 Disease outcomes

The primary outcome was cataract of any type; entries of cataract surgery reimbursement codes were deemed to be a reliable proxy for a previous cataract diagnosis and were included on equal terms as cataract diagnosed by investigators. Studies in which cataract had not been diagnosed by clinical examination, either directly in the studies or indirectly by reimbursement codes in insurance claim registries, were excluded.

### 2.5 Data abstraction and quality assessment

We used Covidence (Covidence systematic review software, Veritas Health Innovation, Melbourne, Australia) to manage references. One reviewer (S.S.) removed duplicates from the literature search. Two reviewers (S.S. and J.B.) selected studies from titles and abstracts. Papers were then selected following full‐text assessment by two reviewers (S.S. and J.B.). From the final sample of papers, two reviewers (S.S. and J.B.) extracted data on study characteristics, design, cataract prevalence or cases and, if available, adjusted risk estimates. Two authors (S.S. and J.B.) assessed the risk of bias in the included studies using The Newcastle‐Ottawa Scale (NOS) (Wells et al., n.d.). A third reviewer (J.P.E.B.) was invited to resolve disagreement between initial reviewers at any point.

### 2.6 Data analysis and synthesis

If risk estimates for the association between welding exposure and cataract were not provided, we calculated the estimates from available data. Heterogeneity was quantified with *I* ^2^ statistics (Higgins et al., 2019). Haldane‐Anscombe correction was used in OR calculation if cataract cases were zero in either the welder or control groups. Funnel plots were used to evaluate the risk of publication bias across studies (Egger et al., 1997). When possible, we used the OR from adjusted analyses. Meta‐analyses were performed with a random‐effects model with pooled OR of the risk of cataract in welders compared to controls in selected subgroups of studies. All statistical analyses were performed using R4.3.0 (R Core Team, 2023). R: A Language and Environment for Statistical Computing. R Foundation for Statistical Computing, Vienna, Austria. URL https://www.R‐project.org/).

## 3 RESULTS

### 3.1 Data retrieval

**FIGURE 1:**
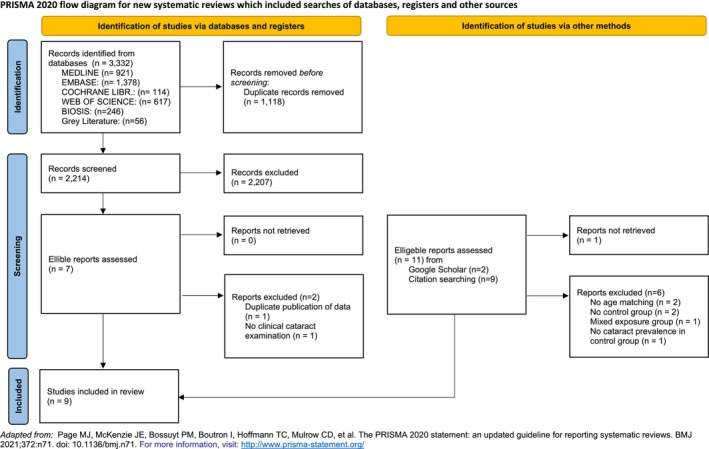
Screening, exclusion and inclusion flow diagram.

A flow chart illustrating the study selection process is included (Figure 1). A total of 2214 records were identified for screening in the formal literature search. Among these, 7 reports concerning welding and cataract were retrieved. Eleven additional studies were found by informal searching of citations and suggestions in Google Scholar, of which one study could not be retrieved in full text. In total, 17 publications were reviewed in full text, 8 of which were discarded for lack of age matching or information on age (*n* = 2), lack of control group (*n* = 2), exposure group including other than welders (*n* = 1), no information on cataract prevalence or cases in control group (*n* = 1), duplicate publication of data (*n* = 1), or cataract diagnosis by interview and not clinical examination (*n* = 1). Consequently, nine studies were found eligible for inclusion (Figure 1), which summarized data of 5165 welders and 513 026 controls.

One large study contributed the majority of welders (*n* = 4288; 83%) (Slagor et al., 2016), as well as cataract cases among welders (*n* = 266; 64%). The second largest study contributed 5% of welders (*n* = 276) and 20% of the cataract cases among welders (*n* = 82) (Bhumika et al., 2014). All remaining studies each contributed less than 3% of total welders and less than 5% of total cataract cases among welders (Alexander et al., 2016; Bochow et al., 1989; Davies et al., 2007; Emmett et al., 1981; Kumari et al., 2016; Megbele et al., 2012; Praveena et al., 2022).

### 3.2 Study characteristics

Four of the identified studies were conducted in India (Alexander et al., 2016; Bhumika et al., 2014; Kumari et al., 2016; Praveena et al., 2022), two in Nigeria (Davies et al., 2007; Megbele et al., 2012), two in USA (Bochow et al., 1989; Emmett et al., 1981) and one in Denmark (Slagor et al., 2016). The studies were conducted from 1981 to 2016. When categorizing the studies by income status of the study countries and geographical area according to the World Bank country classifications by income level (2022–2023), in which countries are stratified into four categories based on Gross National Income (GNI) per capita (low income; lower‐middle income; upper‐middle income; high income) (The World Bank, 2022), six studies were conducted in lower‐middle‐income countries (Alexander et al., 2016; Bhumika et al., 2014; Davies et al., 2007; Kumari et al., 2016; Megbele et al., 2012; Praveena et al., 2022), while three were sourced from high‐income countries (Bochow et al., 1989; Emmett et al., 1981; Slagor et al., 2016). One study was a registry‐based cohort study (Slagor et al., 2016), one study a case–control study (Bochow et al., 1989), while all other studies were cross‐sectional studies comparing welders with controls not exposed to welding. Study characteristics can be found in Table 1.

**TABLE 1:** Study characteristics.

Study	Year	Country	Study type	Cataract examination	Cataract definition	Cataract assessor
Alexander et al	2016	India	Cross‐sectional	Clinical examination	NA	NA
Bhumika et al	2014	India	Cross‐sectional	Clinical examination	NA	NA
Bochow et al	1989	US	Case–control	Clinical examination	Posterior subcapsular cataract	Ophthalmic practice
Davies et al	2007	Nigeria	Cross‐sectional	Slit lamp ophthalmoscopy	NA	NA
Emmett et al	1981	US	Cross‐sectional	Slit lamp	Posterior subcapsular cataract or anterior capsular lens opacity	Ophthalmologist
Kumari et al	2016	India	Cross‐sectional	Ophthalmoscopy	≥5% opacity of the lens surface	Ophthalmology department
Megbele et al	2012	Nigeria	Cross‐sectional	Ophthalmoscopy	≥5% opacity of the lens surface	Ophthalmologist
Praveena et al	2022	India	Cross‐sectional	Slid biomicroscopy	NA	Tertiary care hospital
Slagor et al	2016	Denmark	Cohort	NA	Regular and irregular cataract	Ophthalmologist

Abbreviation: NA, not applicable.

### 3.3 Welding exposure

Occupational exposure to welding was assessed by job titles and/or self‐reports on former and current welding activities. No UVR exposure measurements were available in any of the studies. The duration of employment as a welder ranged from 1 to more than 20 years. Some studies did not report the length of employment (Bochow et al., 1989; Kumari et al., 2016; Megbele et al., 2012; Praveena et al., 2022). Welding activities in the included studies spanned over five decades, from the 1950s to the 2010s. Four studies provided no information on safety equipment (Bochow et al., 1989; Davies et al., 2007; Kumari et al., 2016; Slagor et al., 2016), while five studies declared that either safety goggles/glasses (Alexander et al., 2016; Bhumika et al., 2014; Emmett et al., 1981; Praveena et al., 2022) or welding screens/helmets (Bhumika et al., 2014; Emmett et al., 1981; Megbele et al., 2012) had been used by all or most welders. Welding methods were defined as arc welding (Alexander et al., 2016; Bochow et al., 1989; Davies et al., 2007; Emmett et al., 1981; Kumari et al., 2016; Megbele et al., 2012; Slagor et al., 2016), carbide welding (Davies et al., 2007) and all types (Emmett et al., 1981), but were unknown in two studies (Bhumika et al., 2014; Praveena et al., 2022). Two studies specifically stated that welders and controls had been recruited from informal or unorganized workplaces (Alexander et al., 2016; Praveena et al., 2022), and one study recruited welders working along major streets in Nigeria. Details regarding exposure are presented in Table 2.

**TABLE 2:** Welding exposure and control group characteristics.

Study	Welders/cases	Control group	Welding type	Welders, duration of employment	Eye protection (welders group)
Alexander et al	47 unorganized welding units	Roadside vendors and shopkeepers	Metal arc welding (93%) Gas metal arc welding (7%)	<10 years: 34% 10–20 years: 33% >20 years: 33%	61% used safety glasses
Bhumika et al	Welders in the shipbuilding industry	Workers in the shipbuilding industry not exposed to welding	NA	20 years (SD 12), range 1–40	Welding screen or goggles
Bochow et al	Posterior subcapsular opacities and cataract extraction	Controls from the same practice without posterior subcapsular opacities	Arc welding	NA	NA
Davies et al	Welders working along the major streets	Healthy males	Arc welding (82%) Carbide welding (18%)	8 years (SD 9)	NA
Emmett et al	Fabrication facility	Other workers at the same facility	All types of welding, predominantly arc welding	17 years	Light calibre tint in helmet or safety glasses
Kumari et al	Various sites	Healthy males	Metal arc welding	≥1 year	NA
Megbele et al	Workers at five metal fabrication companies	Other workers at the same companies	Arc welding	NA	Helmets
Praveena et al	Welders at unorganized workplaces	Mostly street vendors and shopkeepers	NA	≥2 years	Goggles
Slagor et al	Welders at 75 industrial workplaces	Skilled and unskilled male workers	Arc welding	≤10 years: 41% 11–20 years: 31% >20 years: 29%	NA

### 3.4 Cataract definition

Definition of cataract were either non‐explicit (Alexander et al., 2016; Bhumika et al., 2014; Davies et al., 2007; Praveena et al., 2022) to ‘≥5% opacity of the lens surface’ (Kumari et al., 2016; Megbele et al., 2012), ‘posterior subcapsular cataract’ (Bochow et al., 1989; Emmett et al., 1981), ‘anterior capsular opacity' (Emmett et al., 1981) or ‘regular cataract’ (ordinary cataract and cataract surgery) as opposed to ‘irregular cataract’ (known causes such as trauma, juvenile/congenital, diabetes or other illness or medication) (Slagor et al., 2016). Cataract examination and assessors were not defined in most studies, but examination explicitly included slit‐lamp or ophthalmoscopy in 5 studies (Davies et al., 2007; Emmett et al., 1981; Kumari et al., 2016; Megbele et al., 2012; Praveena et al., 2022). In 6 studies, cataract assessment was undertaken by an ophthalmologist (Emmett et al., 1981; Megbele et al., 2012; Slagor et al., 2016) or at an ophthalmic practice or hospital (Bochow et al., 1989; Kumari et al., 2016; Praveena et al., 2022; Slagor et al., 2016).

### 3.5 Cataract among welders

Cataract prevalence or cases ranged from 1% to 30% among welders and 0–12% among controls in the cross‐sectional and cohort studies (Table 3). Two studies reported risk estimates adjusted for a number of variables in addition to age: Slagor et al. (2016) reported a hazard ratio (HR), adjusted for age, diabetes, and social group, of 1.08 (95% CI 0.95–1.22), while Megbele et al. (2012) reported an OR, adjusted for age, smoking status, family history of cataract, mainly outdoor work, and history of eye injury, of 3.49 (95% CI 0.37–32.7). In the remaining studies, only crude ORs were available, but the studies controlled for age at the inclusion level, as the investigators noted no significant differences in age between welders and controls.

**TABLE 3:** Cataract outcomes among exposure and control groups.

Study	Welders, mean age	Controls, mean age	Welders no.	Welders, cataract no.	Welders, cataract %	Controls no.	Controls, cataract no.	Controls, cataract %	Demographic comparison or matching
Alexander et al	36 (SD 13)	37 (SD 11)	150	17	11	150	5	3	Age groups, smoking status, alcohol consumption
Bhumika et al	43 (SD 11)	42 (SD 11)	276	82	30	276	29	11	Age, socioeconomic status, smoking status
Bochow et al	NA	NA	^a^	20	^a^	^a^	11	^a^	Age, sex
Davies et al	28 (SD 10)	28 (SD 9)	110	3	3	85	0	0	Age
Emmett et al	43 (SD 1)	44 (SD 1)	77	1	1	58	1	2	Smoking status, sun exposure
Kumari et al	40 (SD 10)	36 (SD 8)	37	3	9	100	1	1	Age groups, sex
Megbele et al	36 (SD 10)	36 (SD 9)	117	9	8	105	1	1	Age groups, sex, smoking status
Praveena et al	33 (SD 12)	33 (SD 11)	90	13	14	90	11	12	Age, socioeconomic status
Slagor et al	41 (SD 10)	40 (SD 12)	4288	266	6	512 151	29 007	6	Age, diabetes mellitus, social group

Abbreviations: NA, not applicable; no., number; SD, standard deviation.

^a^ Case–control study.

### 3.6 Meta‐analysis findings

**FIGURE 2:**
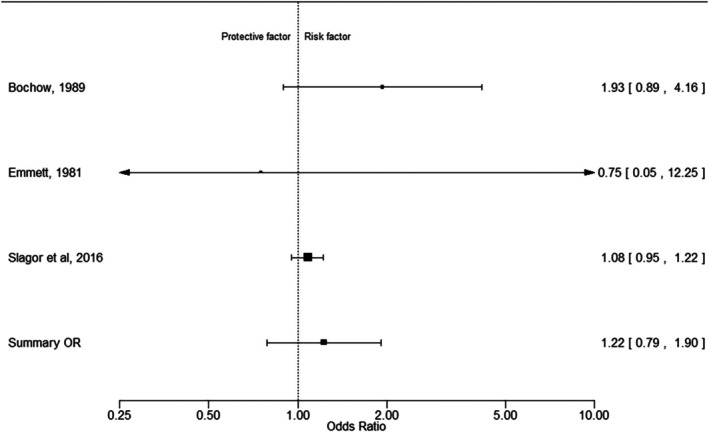
Forest plot and meta‐analysis of studies conducted in high‐income countries. Risk estimates for cataract among welders (hazard ratio from Slagor et al. and odds ratios from other studies). Adjusted risk estimate is shown from Slagor et al. whereas crude risk estimates are presented in remaining studies.

**FIGURE 3:**
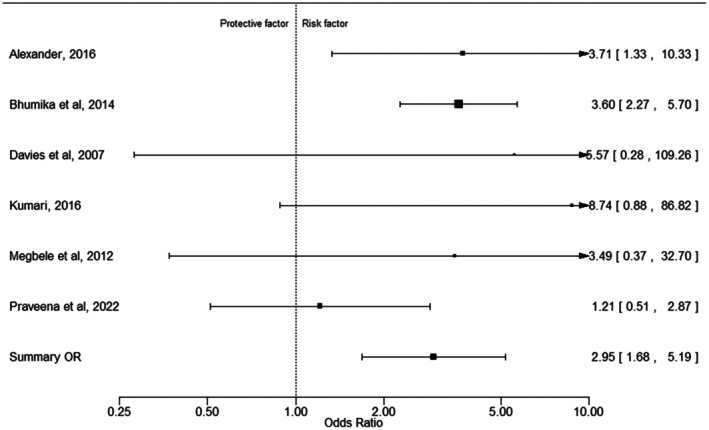
Forest plot and meta‐analysis of studies conducted in lower‐middle income countries. Odds ratios for cataract among welders. Adjusted risk estimate is shown from Megbele et al. whereas crude risk estimates are presented from remaining studies.

**FIGURE 4:**
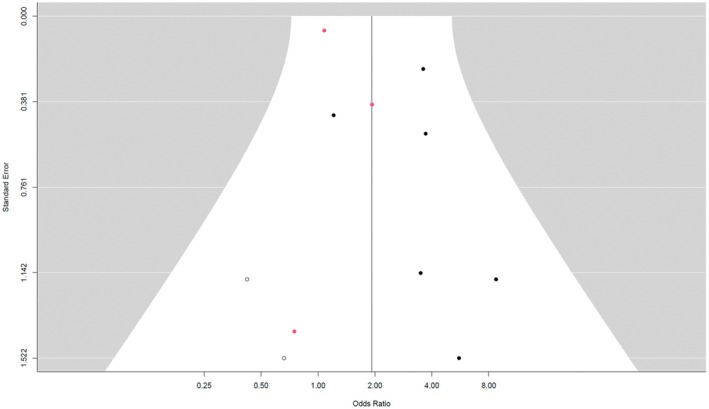
Funnel plot. Funnel plot showing asymmetry. Red circles: Studies from high‐income countries. Black circles: Studies from low‐income countries. White circles: Possible missing studies.

Heterogeneity statistics in exploratory meta‐analysis including all studies showed substantial heterogeneity (*I* ^2^ = 39). For this reason, we judged that a meta‐analysis of all studies combined was not merited. Subgroup meta‐analyses stratified by income status of study countries were therefore undertaken, in which a summary estimate OR of 1.22 [95% CI 0.79–1.90] (*p* = 0.374) was found for cataract among welders in studies conducted in high‐income countries (Figure 2), and a summary estimate OR of 2.95 [95% CI 1.68–5.19] (*p* = 0.00017) was found in analysis of studies originating from lower‐middle‐income countries (Figure 3). The funnel plot showed a possible right‐skewed distribution of studies, which may indicate publication bias in favour of higher risk estimates (Figure 4).

### 3.7 Risk of bias assessment

Newcastle‐Ottawa Scale study quality scores were generally mediocre, mainly due to inadequate comparability between exposed and control groups, specifically lack of adjustment for covariates at the analysis level, including age, diabetes, smoking and previous eye trauma, which could result in bias with an unpredictable direction. Missing information on years in the welding profession or inclusion of younger individuals raised concerns if duration of welding was sufficient to allow for cataract to develop (‘Outcome item #2’). No statement regarding adherence to assigned sampling and allocation reduced scores in most studies (‘Outcome item #3’). The highest study quality scores were found for Slagor et al. (2016) (8 of 9 points) and Megbele et al. (2012) (7 of 9), whereas the lowest were found for Kumari et al. and Davies et al. (both 2 of 9) (Kumari et al., 2016; Megbele et al., 2012). Study quality scores can be found in Table 4.

**TABLE 4:** Risk of bias assessment of studies. Study quality of included studies using the Newcastle‐Ottawa Quality Assessment Scale.

References	Selection				Comparability	Outcome			Quality score
	#1	#2	#3	#4	#1	#1	#2	#3	
	[0–1 ★]	[0–1 ★]	[0–1 ★]	[0–1 ★]	[0–2 ★]	[0–1 ★]	[0–1 ★]	[0–1 ★]	[0–9 ★]
Alexander et al.	0	1	1	0	0	1	1	0	4
Bochow et al.*	1	1	0	1	1	1	1	0	6
Bhumika et al.	1	1	1	0	0	1	1	0	5
Davies et al.	0	0	1	0	0	1	0	0	2
Emmett et al.	1	1	1	0	0	1	1	0	5
Kumari et al.	0	0	1	0	0	1	0	0	2
Megbele et al.	1	1	1	0	2	1	0	1	7
Praveena et al.	0	1	1	0	0	1	0	0	3
Slagor et al.	1	1	1	0	2	1	1	1	8

*Note*: The Newcastle‐Ottawa Quality Assessment Scale (NOS) evaluates study quality within three domains: Selection, Comparability, and Outcome. Two adapted versions of the NOS scale are used to evaluate cohort studies (in this case used for cross‐sectional studies) and case–control studies, respectively. Within the Selection domain, the evaluation categories encompass (#1) the representativeness of the exposed cohort, (#2) the selection methodologies for the non‐exposed cohort, (#3) the ascertainment of exposure, and (#4) the verification that the outcome of interest was not present at the initiation of the study. In the domain of Comparability, the critical assessment focuses on the (#1) comparability of cohorts based on the design or analytical approach. For the Outcome domain, the criteria include (#1) the methodology for outcome assessment, (#2) the adequacy of the follow‐up duration to allow for the occurrence of outcomes, and (#3) the comprehensiveness of cohort follow‐up. A maximum of two points (★) can be awarded within the comparability item, whereas a maximum of one point can be awarded for other items. The cumulative quality score is a summation of the points accrued across all evaluated categories within each study. *The NOS Case–Control Study version was used (Cohort Study version used for all other studies).

## 4 DISCUSSION

This systematic review explored the association between metal welding and cataract in the available medical literature. We found a limited number of highly heterogeneous studies. Subgroup meta‐analysis found a statistically significant increased risk of cataract among welders in six studies carried out in lower‐middle‐income countries, while a slightly increased risk with confidence limits including null was found in three studies performed in high‐income countries. Welding UVR exposure levels are likely to differ with differences in country income, as workers in lower‐income countries may experience limited safety standards and less access to advanced welding safety technology (Ncube & Kanda, 2018). Consequently, UVR exposure levels may be systematically higher in the studies performed in low‐ or lower‐middle‐income countries, and the same direction of effects might also be found for other types of potentially cataractogenous exposures at workshops, such as infrared exposure (Söderberg et al., 2016) or welding fumes and poor ventilation conditions. In addition, welders in two of the included studies from lower‐middle‐income countries were sampled from unorganized workplaces (Alexander et al., 2016; Praveena et al., 2022), in which safety measures mandated by local occupational health jurisdiction have probably not been enforced adequately.

It should be noted that the studies from lower‐middle‐income countries were undertaken in India and Nigeria, which are located closer to the equator than the USA and Denmark, from which the high‐income‐country studies originated. A higher accumulated solar UV exposure could therefore explain part of the higher prevalence of cataract in these regions (Sasaki et al., 2003), but is unlikely to confound the association between welding and cataract in studies where both welders and controls were drawn from similar environments. Solar UV exposure is a determinant for cataract but probably not directly associated with the occupation of welding, and is therefore unlikely to act as a confounder. However, previous or cumulative solar UVR exposure may modify the effect of welding‐related UVR — a potential ‘first and second hit’ mechanism, where pre‐existing lens damage from chronic solar exposure increases susceptibility to further cataract formation from additional UVR during welding.

No studies included direct measurement of UVR exposure, which involves radiometric, spectroradiometric, or personal dosimetry methods (Tenkate, 2017; Vecchia et al., 2007). Nor did the studies distinguish between solar and welding‐related UV sources. This limits the ability to assess welding‐specific UVR effects and potential interactions between solar and welding exposure. Instead, occupational exposure to welding was defined using proxy measures, predominantly as *ever* versus *never welded*. The length of employment as a welder was reported in some of the studies and varied significantly. Dose–response calculations were performed by one study with no statistically significant findings (Slagor et al., 2016). Cumulative exposure, both within and across study populations, varied considerably, disabling direct comparison. Only one study considered a potential time lapse between exposure and the onset of cataract development (Slagor et al., 2016). Several studies included younger individuals (<40 years) and/or welders with limited years of welding experience, which may not be adequate to study cataract development, as both duration of exposure and age influence cataract formation (Allen, 2011). It cannot be ruled out, however, that even young welders with limited access or adherence to modern welding safety equipment could develop cataract within a few years due to excessively high exposure.

There were differences across studies regarding welding methods, with arc welding being the most predominant type. The highest emission of UV radiation occurs in gas metal arc welding (‘American Welding Society (2014). Safety and health fact sheet No. 26: Arc viewing distance.’ n.d.). However, workers are less inclined to wear eye protection in other forms of metal welding (Burgess, 1995; Peng et al., 2007). It is therefore uncertain how the different types of welding affected the overall results.

We identified two studies (Bochow et al., 1989; Emmett et al., 1981) that categorized cataracts into one of the three major types of age‐related cataract (nuclear, cortical, and posterior subcapsular cataract). Both studies reported no significant difference in the prevalence or number of cases of posterior subcapsular cataract between welders and controls. The remaining studies either used cataract grading schemes not recommended by the WHO (Kumari et al., 2016; Megbele et al., 2012) or did not disclose which cataract grading systems were used (Alexander et al., 2016; Bhumika et al., 2014; Davies et al., 2007; Praveena et al., 2022; Slagor et al., 2016). In addition, more than half of the studies did not specifically state that ophthalmologists undertook the cataract examination and grading, with only one study from the lower‐middle‐income country group explicitly stating that assessment was done by an ophthalmologist (Megbele et al., 2012). Thus, a systematic bias regarding the outcome might be present primarily in the lower‐middle‐income country subgroup, and findings must be interpreted with caution.

Only two studies provided risk estimates adjusted for possible confounders at the analysis level, both with statistically insignificant risk estimates (Megbele et al., 2012; Slagor et al., 2016). A statistically significant association between welding and cataract was reported in two studies, which both originated from India and provided crude risk estimates only (Alexander et al., 2016; Bhumika et al., 2014). Lack of adjusted analyses might have contributed to our finding of a statistically significant summary risk estimate for cataract among welders found in the subgroup analysis of studies from lower‐middle‐income countries. Megbele et al. was the only study that controlled for a history of eye injuries, which primarily had occurred at work (Megbele et al., 2012). The findings indicated that eye trauma at work was the main occupational risk factor for cataract among welders. The use of protective measures against eye injuries, which may prevent traumatic cataract, likely differs between workplaces and countries.

The remaining studies had controlled for age at the sampling level, and some also described prevalences of other risk factors for cataract, such as smoking (Alexander et al., 2016; Bhumika et al., 2014; Emmett et al., 1981; Megbele et al., 2012), alcohol consumption (Alexander et al., 2016), diabetes mellitus (Kumari et al., 2016; Megbele et al., 2012; Slagor et al., 2016), and sunlight exposure (Emmett et al., 1981, Megbele et al., 2012). Only one study accounted for prior use of systemic corticosteroid treatment (Megbele et al., 2012). Residual confounding may vary in direction and is unpredictable across studies.

Ultimately, the correlation between welding and cataract is biologically feasible, as UVR is widely recognized as a possible risk for cataract (Borges‐Rodríguez et al., 2023). Welding might be a risk factor for cataract when welding is performed without adequate protection throughout the welding career. Nevertheless, the literature summarized in this review does not provide strong evidence that welding is increasing the risk of cataract, although this is highly biologically plausible. To provide further evidence, large studies in various and well‐defined exposure settings with adjusted analyses are needed, including with quantitative or modelled UVR dosimetry, adjusted for latitude and outdoor work, and, where possible, distinguish between occupational and ambient UVR contributions.

### 4.1 Strengths and limitations

Strengths of the study include a comprehensive literature search across several medical literature databases as well as a pre‐published protocol signalling our intentions. The study was reported following PRISMA guidelines (Page et al., 2021).

Limitations include lack of information in several studies regarding exposure variables that might have influenced cataract formation among welders as well as UVR exposure measurements, types of welding done by welders, whether protective equipment was used and, if so, their type, and years spent working in the welding profession. In addition, we found a considerable heterogeneity among available studies regarding (likely) use of personal protective equipment, information regarding cataract assessment methods and health care professionals employed to assess cataract, as well as sample sizes, with the study by Slagor et al. carrying a disproportional weight in the analysis. These factors present challenges in synthesizing data and drawing generalized conclusions, underscoring the need for cautious interpretation of the findings. The varying definitions of cataract found in the studies may lead to misclassification, thereby restricting the comparability of cataract between studies. Publication bias indicated by the Funnel plot should be interpreted with caution due to the small number of studies available. Despite these limitations, this systematic review provides valuable insights into occupational health risks for metal welders and highlights the critical need for standardized methodologies in future research to better understand and mitigate the risks of cataract development in the welding profession.

## 5 CONCLUSIONS

The included studies differed in design, populations, and confounder control as well as exposure and outcome assessment. Nevertheless, a consistent association between occupational welding exposures and cataract in lower‐middle‐income countries was shown, while studies from high‐income populations yielded smaller, non‐significant associations. These findings could be due to inadequate welding safety adherence with insufficient welding‐related UVR exposure protection in lower‐middle‐income countries. Larger studies with harmonized methodologies are needed to quantify exact effect sizes in the possible association between metal welding and cataract.

## FUNDING INFORMATION

Arbete och Hälsa, Occupational and Environmental Medicine, University of Gothenburg, Sweden and Department of Occupational and Environmental Medicine, Bispebjerg Frederiksberg Hospital, Copenhagen, Denmark (no grant number specified by the funders).

## CONFLICT OF INTEREST STATEMENT

The authors declare no commercial relationship with suppliers of ophthalmic therapeutics or devices and no other conflict of interest.

## APPENDIX A

### A.1

**TABLE A1:** Literature search.

Database or register	Search	Hits
Pubmed	((((“outdoor”[All Fields] OR “outdoors”[All Fields]) AND (“Work”[MeSH Terms] OR “Work”[All Fields] OR “job”[All Fields] OR (“occupant”[All Fields] OR “occupant s”[All Fields] OR “occupants”[All Fields] OR “occupational”[All Fields] OR “Occupations”[MeSH Terms] OR “Occupations”[All Fields] OR “occupation”[All Fields]))) OR (“pilots*”[All Fields] OR “flight personnel”[All Fields] OR “cabin crew”[All Fields] OR “astronaut*”[All Fields] OR (“united states national aeronautics and space administration”[MeSH Terms] OR (“united”[All Fields] AND “states”[All Fields] AND “national”[All Fields] AND “aeronautics”[All Fields] AND “space”[All Fields] AND “administration”[All Fields]) OR “united states national aeronautics and space administration”[All Fields] OR “nasa”[All Fields]) OR “radium dial painter”[All Fields] OR (“chernobyl”[All Fields] OR “chernobyl s”[All Fields]) OR “Mayak”[All Fields] OR “air force”[All Fields] OR “navy”[All Fields] OR “urologist*”[All Fields] OR “anesthesiologist*”[All Fields] OR “anesthetist*”[All Fields] OR “cardiologist*”[All Fields] OR “urologist*”[All Fields] OR “endoscopist*”[All Fields] OR “orthopaedic*”[All Fields] OR “radiologic technologist*”[All Fields] OR “nuclear medicine personnel”[All Fields] OR “dental worker*”[All Fields] OR “dentist*”[All Fields] OR “dental staff”[All Fields] OR “radiographer*”[All Fields] OR “radiologist*”[All Fields] OR “metal worker*”[All Fields] OR “welder*”[All Fields] OR “silviculturist*”[All Fields] OR “horticulturist*”[All Fields] OR “farm worker*”[All Fields] OR “gardener*”[All Fields] OR “park worker*”[All Fields] OR “postmen”[All Fields]) OR (“Occupational Diseases”[MeSH Terms] OR “Occupational Exposure”[MeSH Terms] OR “Occupational Health”[MeSH Terms] OR “Occupations”[MeSH Terms] OR “Work”[MeSH Terms])) AND (“Cataract”[MeSH Terms] OR “cataract*”[Text Word])) AND ((humans[Filter]) AND (danish[Filter] OR english[Filter] OR norwegian[Filter] OR swedish[Filter]))	921
Embase	(“occupational disease” or “occupational health” or occupation* or pilots* or “flight personnel” or “cabin crew” or astronaut* or NASA or “radium dial painter” or Chernobyl or Mayak or “air force” or “navy” or Urologist* or Anesthesiologist* or anesthetist* or cardiologist* or Urologist* or endoscopist* or orthopaedic* or “radiologic technologist*” or “nuclear medicine personnel” or “dental worker*” or dentist* or “dental staff” or radiographer* or radiologist* or “metal worker*” or welder* or silviculturist* or horticulturist* or “farm worker*” or gardener* or “park worker*” or postmen or “Outdoor work*”).mp. [mp = title, abstract, heading word, drug trade name, original title, device manufacturer, drug manufacturer, device trade name, keyword heading word, floating subheading word, candidate term word] AND exp. cataract/ or cataract*.mp. [mp = title, abstract, heading word, drug trade name, original title, device manufacturer, drug manufacturer, device trade name, keyword heading word, floating subheading word, candidate term word] LIMIT 30 to (human and (danish or english or norwegian or swedish))	1378
Cochrane	#1 (cataract*):ti,ab,kw (Word variations have been searched) #2 MeSH descriptor: [Cataract] explode all trees #3 #1 OR #2 #4 MeSH descriptor: [Occupational Diseases] explode all trees #5 pilots* OR (flight NEXT personnel) OR “cabin crew” OR astronaut* OR NASA OR (radium NEXT dial NEXT painter) OR Chernobyl OR Mayak OR “air force” OR “navy” OR Urologist* OR Anesthesiologist* OR anesthetist* OR cardiologist* OR Urologist* OR endoscopist* OR orthopaedic* OR (radiologic NEXT technologist*) OR “nuclear medicine personnel” OR (dental NEXT worker*) OR dentist* OR “dental staff” OR radiographer* OR radiologist* OR (metal NEXT worker*) OR welder* OR silviculturist* OR horticulturist* OR (farm NEXT worker*) OR gardener* OR (park NEXT worker*) OR postmen OR (Outdoor NEXT work*) #6 MeSH descriptor: [Occupational Exposure] explode all trees #7 MeSH descriptor: [Occupational Health] explode all trees #8 MeSH descriptor: [Accidents, Occupational] explode all trees #9 MeSH descriptor: [Occupational Injuries] explode all trees #10 MeSH descriptor: [Occupations] explode all trees #11 MeSH descriptor: [Work] explode all trees #12 #4 OR #5 OR #6 OR #7 OR #8 OR #9 OR #10 OR #11 #13 #3 AND #12	114
Web of science	(“Occupational Exposure” OR “work exposure” OR pilots* OR “flight personnel” OR “cabin crew” OR astronaut* OR NASA OR “radium dial painter” OR Chernobyl OR Mayak OR “air force” OR navy or Urologist* OR Anesthesiologist* OR anesthetist* OR cardiologist* PR urologist* OR endoscopist* OR orthopaedic* OR “radiologic technologist*” OR “nuclear medicine personnel” OR “dental worker*” OR dentist* OR “dental staff” OR radiographer* OR radiologist* OR “metal worker*” OR welder* OR silviculturist* OR horticulturist* PR “farm worker*” OR gardener* OR “park worker*” OR postman OR “outdoor work*”) AND ((TI = (cataract*)) OR AK = (cataract*)) OR AB = (cataract*) AND English (Languages)	617
BIOSIS	TS = (“Occupational Exposure” OR “work exposure” OR pilots* OR “flight personnel” OR “cabin crew” OR astronaut* OR NASA OR “radium dial painter” OR Chernobyl OR Mayak OR “air force” OR navy or Urologist* OR Anesthesiologist* OR anesthetist* OR cardiologist* PR urologist* OR endoscopist* OR orthopaedic* OR “radiologic technologist*” OR “nuclear medicine personnel” OR “dental worker*” OR dentist* OR “dental staff” OR radiographer* OR radiologist* OR “metal worker*” OR welder* OR silviculturist* OR horticulturist* PR “farm worker*” OR gardener* OR “park worker*” OR postman OR “outdoor work*”) AND ((TI = (cataract*)) OR AB = (cataract*)) OR MC = (cataract*) AND English (Languages)	246
Open Grey	“cataract”	56

*Note*: Search terms from the formal literature search.
